# Classification and Characterization of Species within the Genus *Lens* Using Genotyping-by-Sequencing (GBS)

**DOI:** 10.1371/journal.pone.0122025

**Published:** 2015-03-27

**Authors:** Melissa M. L. Wong, Neha Gujaria-Verma, Larissa Ramsay, Hai Ying Yuan, Carolyn Caron, Marwan Diapari, Albert Vandenberg, Kirstin E. Bett

**Affiliations:** Department of Plant Sciences, University of Saskatchewan, 51 Campus Drive, Saskatoon, SK, S7N 5A8, Canada; Agriculture and Agri-Food Canada, CANADA

## Abstract

Lentil (*Lens culinaris* ssp. *culinaris*) is a nutritious and affordable pulse with an ancient crop domestication history. The genus **Lens** consists of seven taxa, however, there are many discrepancies in the taxon and gene pool classification of lentil and its wild relatives. Due to the narrow genetic basis of cultivated lentil, there is a need towards better understanding of the relationships amongst wild germplasm to assist introgression of favourable genes into lentil breeding programs. Genotyping-by-sequencing (GBS) is an easy and affordable method that allows multiplexing of up to 384 samples or more per library to generate genome-wide single nucleotide Polymorphism (SNP) markers. In this study, we aimed to characterize our lentil germplasm collection using a two-enzyme GBS approach. We constructed two 96-plex GBS libraries with a total of 60 accessions where some accessions had several samples and each sample was sequenced in two technical replicates. We developed an automated GBS pipeline and detected a total of 266,356 genome-wide SNPs. After filtering low quality and redundant SNPs based on haplotype information, we constructed a maximum-likelihood tree using 5,389 SNPs. The phylogenetic tree grouped the germplasm collection into their respective taxa with strong support. Based on phylogenetic tree and STRUCTURE analysis, we identified four gene pools, namely *L*. *culinaris/L*. *orientalis/L*. *tomentosus*, *L*. *lamottei/L*. *odemensis*, *L*. *ervoides* and *L*. *nigricans* which form primary, secondary, tertiary and quaternary gene pools, respectively. We discovered sequencing bias problems likely due to DNA quality and observed severe run-to-run variation in the wild lentils. We examined the authenticity of the germplasm collection and identified 17% misclassified samples. Our study demonstrated that GBS is a promising and affordable tool for screening by plant breeders interested in crop wild relatives.

## Introduction

Lentil (*Lens culinaris* ssp. *culinaris*) is an annual, herbaceous, self-pollinating grain legume crop. This crop is important in cereal-based cropping systems due to its nitrogen-fixing ability. Lentil has a historical and geographic domestication history [1–3]. Lentil and its wild relatives are naturally distributed in South-west Asia and Mediterranean regions [1]. The genus *Lens* (2n = 14) is phylogenetically nested within the tribe Vicieae, which are cool-season legumes belonging to sub-family Papilionoideae of family Fabaceae [4]. The genus *Lens* has seven closely related taxa, namely *L*. *culinaris*, *L*. *orientalis*, *L*. *tomentosus*, *L*. *odemensis*, *L*. *lamottei*, *L*. *ervoides* and *L*. *nigricans*. Past taxonomic studies [5–13], based on morphology, cytogenetics, hybridization studies, and/or molecular markers, have frequently disagreed with respect to classification at the species and subspecies level. The most recent classification identified seven taxa grouped into four species, namely *L*. *culinaris* ssp. *culinaris*, *L*. *culinaris* ssp. *orientalis*, *L*. *culinaris* ssp. *tomentosus*, *L*. *culinaris* ssp. *odemensis*, *L*. *ervoides*, *L*. *lamottei*, and *L*. *nigricans* [14, 15]. Despite the taxonomic re-organizations, all studies generally agreed that *L*. *culinaris* ssp. *orientalis* is the most closely related wild progenitor of *L*. *culinaris* ssp. *culinaris* while *L*. *nigricans* is the most distant relative.

The ancient domestication history of cultivated lentil has produced bottleneck effects resulting in a narrow genetic basis which has resulted in reduced levels of resistance to biotic and abiotic stresses relative to its wild relatives [16]. Phenotypic variability studies have identified wild lentil germplasm with resistance to anthracnose (*Colletotrichum spp*.), ascochyta blight (*Ascochyta lentis*), stemphylium blight (*Stemphylium botryosum*) and *Orobanche* spp. root-holoparasitic infection [17–21]. To increase genetic diversity and resistance to biotic and abiotic stresses in new cultivars, introgression of favourable genes from crop wild relatives is necessary. Like many crops, wild relatives can be divided into primary, secondary and tertiary gene pools, according to their relatedness to *L*. *culinaris* ssp. *culinaris* and their ability to produce fertile hybrids when intercrossed with cultivated lentil [22]. Some early studies, based on cross-compatibility and cytogenetic evidence [23–25], placed *L*. *orientalis* in the primary gene pool while the secondary gene pool consisted of *L*. *odemensis*, *L ervoides* and *L*. *nigricans* [26]. However, the gene pool placement of two more recently identified species *L*. *lamottei* and *L*. *tomentosus* [27] is inconsistent among studies. These two species were placed in the secondary gene pool by Muehlbauer and McPhee [22] while a later study suggested that more crossing experiments would be necessary to determine their positions [28]. In addition, a more recent study suggested that *L ervoides* and *L*. *nigricans* should be placed in the tertiary gene pool [29]. Nonetheless, the gene pools proposed by all these studies are not in concordance with the species classification from the Ferguson et al. study [14] suggesting more work is needed to clarify these relationships.

Successful and efficient diversification of cultivated lentils through introgression of genes from wild relatives greatly depends on accurate identification of the wild species followed by successful development of fertile hybrid plants. Despite having good standard procedures for the management of plant genetic resources, incorrect classification is not uncommon. Furthermore, human error can be introduced at various stages in a breeding program, e.g., seed handling, storage, harvesting and exchange between plant breeders. Some accessions of the wild progenitor *L*. *orientalis* are morphologically almost indistinguishable from the cultivated lentil and the two species are fully inter-fertile [1] making classification difficult. Many studies have employed molecular markers to improve the accuracy of germplasm characterization [30–33], however, the high cost per sample involved in marker discovery and screening has restricted practical application in plant breeding programs. The recent improvements of next-generation sequencing-based genotyping methods has made routine screening of plant germplasm feasible and cost-effective [34].

Genotyping-by-sequencing (GBS) is genome-wide reduced representation of Single Nucleotide Polymorphisms (SNPs) developed for Illumina sequencing technology [35]. Compared to other complexity reduction methods such as Reduced Representation Libraries (RRL) and Restriction site Associated DNA (RAD) sequencing, the GBS method is favoured as a relatively simple and quick method for generating SNP data [36, 37]. The initial protocol was developed using one restriction enzyme [37] and subsequently modified to use two restriction enzymes (a common cutter and a rare cutter) to generate uniform complexity reduction [38]. The two-enzyme approach reduces genome complexity by avoiding sequencing of repetitive regions resulting in more straightforward bioinformatics analysis for large genomes. GBS has already been successfully applied in highly homozygous crops such as maize, rice, soybean, wheat and barley to provide large numbers of SNP markers for association studies and genomic-assisted breeding [37–40]. The low read depth produced by GBS poses a challenge in accurate detection of heterozygous SNPs in mapping populations and crop wild relatives [41, 42]. Nonetheless, recent reports demonstrated the use of GBS in wild crop relatives to resolve phylogenetic relationship and genetic diversity [43–45]. Therefore, the GBS method has great potential for characterization of the large and complex genomes (~4 Gb [46]) of the genus *Lens*.

The objective of this study was to characterize a collection of wild and cultivated *Lens* spp. germplasm currently used in the University of Saskatchewan lentil breeding program to gain a better understanding of taxon and gene pool classification. Here, we explore the use of GBS using a two-enzyme system to discover and genotype genome-wide SNPs. We developed an automated GBS pipeline to facilitate SNP detections from a large number of samples. To improve the understanding of our existing germplasm, we examined the phylogenetic relationships and population structure of the genus *Lens*. We also verified the authenticity of all the accessions and their biological replicates and evaluated the reproducibility of GBS results in species classification and accession identification.

## Materials and Methods

### Plant materials

The genetic diversity panel of the genus *Lens* consisted of 83 samples which originated from 60 diverse varieties and landraces (subsequently known as accessions). As several seed sources may be available for an accession, each seed source represented a biological replicate and was subsequently referred to as a sample. The number of accessions ranged from 5 to 15 for each of the seven taxa. The information about their accession numbers, species classification, seed source and geographical location is available in S1 Table. To obtain plant materials from these accessions, a single plant of each sample was selfed to produce sufficient seeds for the study. About 3–5 seeds were germinated and the seedlings were grown for 2–3 weeks in a controlled environment growth chamber. Genomic DNA extraction was carried out using pooled fresh leaf tissues using a modified CTAB method [47]. The quantity and quality of the genomic DNA was checked on a 1% agarose gel and determined using Quant-iT PicoGreen dsDNA assay kit (Life Technologies, USA) on a FLUOstar Omega fluorometer (BMG Labtech, Germany).

### Library preparation for genotyping-by-sequencing

Two GBS libraries were constructed based on a modified protocol from Poland et al. [38] using a two-enzyme system, PstI (rare cutter) and MspI (common cutter). The protocol consisted of three main steps: restriction digestion, ligation, and PCR amplification. Each GBS library was a 96-plex library consisting of 48 samples in two technical replicates. We first prepared one library consisting of 36 samples from the lentil diversity panel and 12 samples from another experiment. A second GBS library was constructed to include samples from the first library which produced low number of reads and additional samples to increase representation of some species. Each sample was labelled with its accession number as prefix and suffixes representing the seed source (a/b/c/A/S/R) and GBS libraries (r1/r2). See S2 Table for information on GBS libraries.

For each sample, a total of 200 ng of genomic DNA was digested using 8 U of Pst1-HF and 8 U MspI (NEB, USA) in a 20 μl reaction mixture, by incubating at 37 ºC for 2 h followed by denaturation of restriction enzyme at 65 ºC for 20 min. Ligation was performed in a 40 μl reaction volume containing the digested DNA, 0.02 μM (0.1 pM) of Adapter 1 (containing barcode sequence), 3 μM (15 pM) of Adapter 2 and ligation master mix (1X NEB buffer 4, 1 mM ATP and 200 U T4 DNA ligase). The ligation mixture was incubated at 22 ºC for 2 h followed by inactivation of the enzyme at 65 ºC for 20 min. An aliquot of 10 μl of adapter-ligated DNA from each sample was pooled and adjusted to a final volume of 500 μl. About 400 μl of the pooled DNA was purified using QIAquick PCR purification kit (Qiagen, Germany) as per manufacturer's guidelines and eluted into a total volume of 120 μl using EB buffer.

In the amplification step, 8 PCR reactions were set up for each library. Each PCR reaction contained 10 μl of eluted DNA, 1X NEB Master Mix, 0.8 μM Illumina Primer 1 (barcoded adapter with PstI overhangs) and 0.8 μM Illumina Primer 2 (common Y-adapter) prepared in a final volume of 25 μl. PCR amplification was performed using an initial denaturation of 95 ºC for 30 s, followed by 16 cycles of 95 ºC for 30 s, 62 ºC for 20 s and 68 ºC for 30 s and a final elongation step of 72 ºC for 5 min. All eight PCR reactions were pooled and purified using QIAquick PCR purification kit (Qiagen, Germany) and the purified library was eluted in 30 μl EB. The quality and quantity of the library was measured using a Bioanalyzer DNA 1000 Chip (Agilent, USA) and Qubit High Sensitivity dsDNA kit (Invitrogen, USA). The libraries were diluted to 2 nM and sequenced on an Illumina Hiseq 2500 (2 x100 bp) at the DNA Sequencing Laboratory, NRC-Saskatoon. The raw sequencing reads were deposited in NCBI SRA (Accession numbers: SRX703778-SRX703943).

### Read mapping and SNP calling

**Fig 1:**
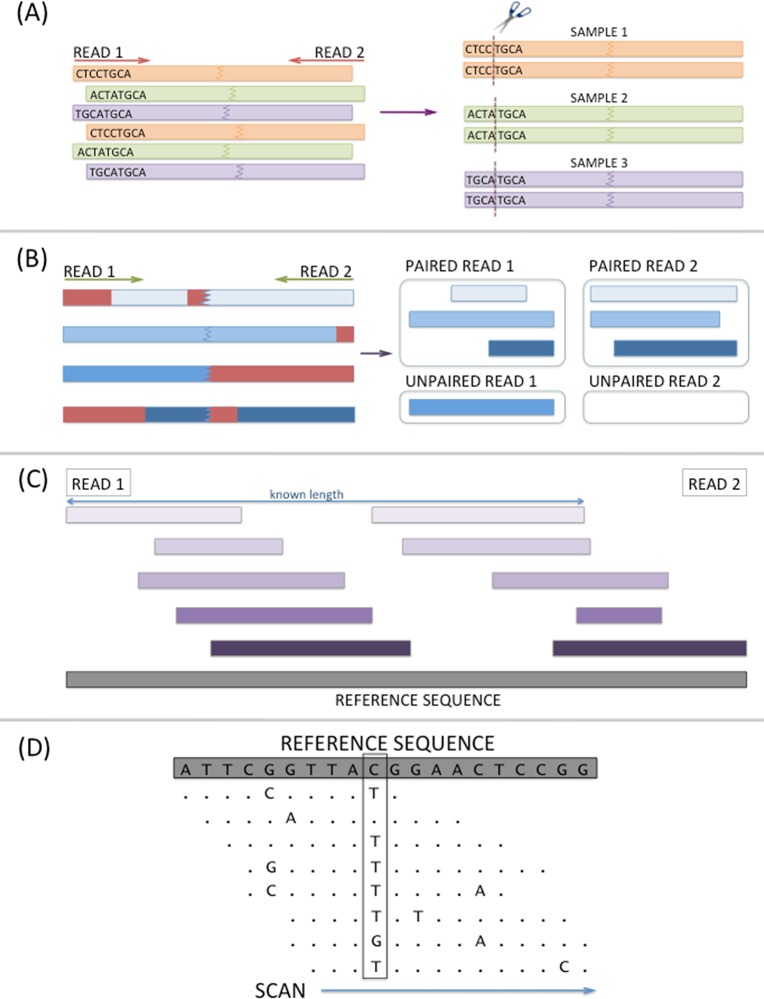
Detailed steps performed by automated GBS pipeline. (A) De-multiplex samples: Raw paired-end Illumina reads are assigned to a sample using barcode sequences, which are subsequently trimmed. (B) Trim and filter reads: De-multiplexed paired-end reads are trimmed for base quality and Illumina adaptors. (C) Align reads to a reference genome. (D) Raw SNP calls: Every position in each sample’s alignment is scanned to determine the probability of a variant.

Raw Illumina reads were de-multiplexed and the barcode sequences removed. Any sequences not containing the expected restriction sites for both enzymes were removed. Subsequently, the reads were filtered and trimmed using recommended settings in Trimmomatic-0.17 [48]. Due to uneven read distribution between the two technical replicates for each sample, both reactions were merged before variant calling. The filtered reads for each sample were aligned to the draft lentil genome (version 0.6) of CDC Redberry (*Lens culinaris*) using Bowtie2-2.1.0 [49] allowing only end-to-end matches. Variant-calling was performed with Samtools-0.1.18 [50] and output in VCF format [51]. The SNP results from all the samples were merged into one large file using custom Perl scripts. This pipeline (Fig 1) is publicly available at http://knowpulse2.usask.ca/pulse-binfo/software/GBS-Pipeline. *In silico* prediction of ApeKI and PstI-MspI fragments was performed on the draft lentil genome (version 0.6) using a custom Python script. In addition, the scaffolds from which the SNPs originated were searched against *M*. *truncatula* genome version Mt4.0 [52] using NCBI-BLAST-2.2.28+[53] to predict genome distribution of SNPs in lentil genome.

### Phylogenetic tree construction

The phylogenetic tree was initially constructed using a relatively easy and quick distance-matrix method until a pipeline with more advanced methods and bootstrapping is available to handle large SNP datasets. For the first GBS run, a pairwise distance matrix was generated based on unfiltered calls across our preliminary test run. After removing samples with a low number of reads, a dendrogram was constructed using the Neighbour-Joining method in Phylip [54] based on this matrix. When the second set of GBS data became available, the phylogenetic tree for both GBS runs was constructed using SNPhylo [55]. The SNP datasets were first filtered for SNPs which fell in repetitive regions detected by Repeatmasker-4.0.3[56] using Repbase-18.11[57] to remove any potential false positive SNPs caused by mismapping. The pipeline was used to construct a maximum-likelihood tree with bootstrap values by performing the following steps: 1) filter the SNP datasets using minor allele frequency of 10% and 20% missing data; 2) remove redundant SNPs based on linkage disequilibrium information (cutoff threshold 0.8) using SNPRelate [58]; 3) construct multiple sequence alignment of the SNP dataset using MUSCLE [59]; 4) construct the maximum-likelihood tree using DNAML from Phylip [54]; 5) perform 1000 bootstraps using Phangorn [60]. The phylogenetic tree was drawn and visualized using iTOL [61].

### Determination of population structure

Determination of population structure was performed on a filtered SNP dataset of all individuals used in maximum-likelihood tree construction using STRUCTURE-2.3.4 [62] based on admixture and the USEPOPINFO model. Linkage disequilibrium was assumed to be absent in the filtered SNP dataset after SNPRelate filtering in the SNPhylo pipeline. The estimation of clusters (K) was performed in five replications using K of 1 to 10. An additional STRUCTURE analysis (K of 1 to 5) was performed on a filtered SNP dataset of individuals from *L*. *odemensis*, *L*. *lamottei*, *L*. *ervoides* and *L*. *nigricans* to evaluate population substructure within these four species. The analysis was optimized using higher burnin period and MCMC Reps after burnin until standard deviations of L(K) were low. The best K value was chosen based on Evanno’s methods [63] using STRUCTURE HARVESTER [64] and visualized using Distruct v1.1 [65].

## Results

The sequencing of the first GBS library consisting of 36 samples resulted in a total of 90,218,745 raw de-multiplexed reads with an average 2.5 million reads per sample. We successfully mapped between 59 and 94% of reads per technical replicate to the reference genome. After excluding two technical replicates from IG_110813_r1 and IG_110810_r1 that yielded less than 1,000 reads, the estimated site coverage per technical replicate ranged from 0.055 to 14.637 x.

As the use of the two-enzyme approach in GBS library construction was selected based on previous reports from other crops which represents *a priori* knowledge in lentil, we evaluated the one-enzyme and two-enzyme GBS approach in our preliminary lentil draft genome assembly using *in silico* prediction of restriction sites. We predicted the restriction sites of ApeKI, MspI and PstI in the lentil draft genome assembly and calculated the number of genomic fragments containing ApeKI-ApeKI (one-enzyme approach) and MspI-PstI (two-enzyme approach) cuts. We found that the two-enzyme MspI-PstI approach is more favourable as it should produce 17% fewer fragments than the ApeKI one-enzyme method. We also evaluated the distribution of 5,389 SNPs used in final phylogenetic tree which were represented in 2,120 scaffolds of the *L*. *culinaris* genome assembly (v0.6) and found them to be evenly distributed across *Medicago truncatula* genome version Mt4.0.

**Fig 2:**
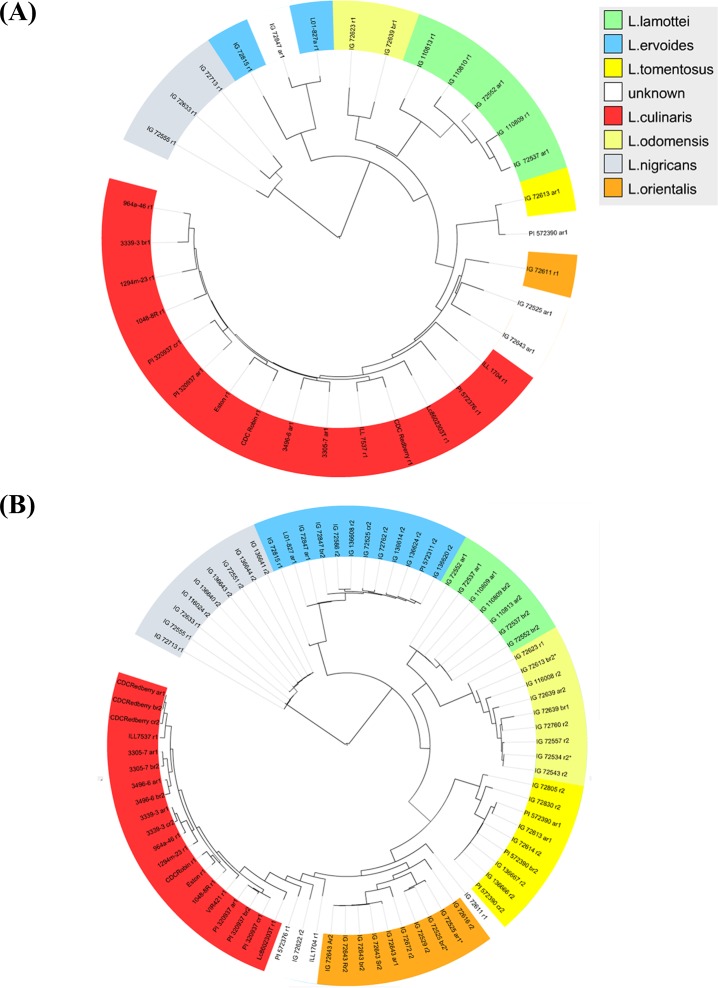
Phylogenetic relationship within genus *Lens*. (A) Dendrogram generated using Neighbour-Joining model based on results from first GBS library. (B) A maximum-likelihood tree based on combined results from two GBS libraries.

A preliminary analysis using Neighbour-Joining tree based on 353,656 unfiltered SNPs classified the diverse germplasm into four major groups; namely *L*. *nigricans*, *L*. *ervoides*, *L*. *odemensis*/*L*. *lamottei*, and *L*. *culinaris/L*. *orientalis/L*. *tomentosus* (Fig 2A). All but four of the samples fell within their respective groups as anticipated. IG 72847 was classified as *L*. *orientalis*, however, it grouped with *L*. *ervoides* IG 72815. Fiala et al. [66] reported that the original seed source of IG 72847 was a seed mixture of *L*. *orientalis* and *L*. *ervoides* since L01-827A, a single-plant selection from IG 72847, also grouped in *L*. *ervoides* and therefore, the sample we used is likely originated from a *L*. *ervoides* plant. PI 572390 was listed as *L*. *orientalis* but it appeared to be *L*. *tomentosus*. Two samples, IG 72525 and IG 72643, grouped with a *L*. *orientalis* accession (i.e. IG 72611) despite being classified as *L*. *ervoides* and *L*. *tomentosus*, respectively. The sterility issues that arose in two populations made from putative intra-specific crosses: IG 72611 x PI 572390 (supposedly a *L*. *orientalis* cross) and IG 72643 x IG 72613 (supposedly a *L*. *tomentosus* cross) [67] provided further evidence that there had been mislabeling or misclassification of the parental accessions IG 72643 and PI 572390.

To verify the authenticity of these four samples, we constructed a second GBS library using old and fresh DNA samples (from different seed sources if available) of 15 accessions from the first GBS library and included 27 additional accessions to improve separation and classification of the wild species during phylogenetic tree construction. Both GBS libraries produced an average of 2.4 million raw de-multiplexed reads per sample. Although the range of reads that mapped to reference genome remained the same, the estimated site coverage was increased to the range of 0.55 to 31.80 x after removing three samples with low number of reads (IG_110813_r1, IG_110810_r1 and IG_72847_cr2). After removing SNPs that were present in repetitive regions of the genome, we detected a total of 266,356 SNPs with 11,581–116,510 SNPs per sample. Removal of SNPs and samples with more than 20% missing SNP data resulted in a final dataset of 32,019 SNPs from 80 samples. Only 0.57% of the SNPs had heterozygous genotypes, mainly found in *L*. *nigricans* and *L*. *ervoides* samples.

A major problem we observed from the sequencing results of both GBS libraries was the large variation in the number of reads assigned to a given barcode adaptor in each library. By having two technical replicates per sample and each technical replicate multiplexed with a different barcode adaptor, we were able to examine whether technical replicate and barcode sequence influenced the number of reads per sample. We found no significant difference in the number of reads per technical replicate between both runs, suggesting that this bias was not caused by run-to-run variation (paired t-test, p>0.05). We also observed that the number of reads were the same between the two technical replicates (paired t-test, p<0.05). Assuming the number of reads between technical replicates was the same, the huge variation of number of reads must be sample dependent and thus, the DNA quantity and quality of the samples was likely to be the main contributing factor to the observed biases.

After removing 893 low quality and 25,737 redundant SNPs, the number of SNPs was reduced to 5,389 and subsequently used to construct a maximum-likelihood tree (Fig 2B). The phylogenetic tree provided 100% bootstrap support for the paraphyletic relationship among the four major *Lens* groups; namely *L*. *nigricans*, *L*. *ervoides*, *L*. *lamottei*/*L*.*odemensis*, and *L*. *culinaris/L*. *orientalis/L*. *tomentosus*. As expected, *L*. *nigricans* exhibited the greatest genetic distance from *L*. *culinaris* followed by *L*. *ervoides*, *L*. *lamottei*/*L*. *odemensis* and finally *L*. *tomentosus*/ *L*. *orientalis* was the closest. The *L*. *lamottei*/*L*. *odemensis* group was further separated into their respective taxa. The *L*. *culinaris/L*. *orientalis/L*. *tomentosus* clade is paraphyletic in which *L*. *tomentosus* first branched off with good bootstrap support followed by the branching of *L*. *orientalis* from *L*. *culinaris* with weak bootstrap support. The species boundary between *L*. *culinaris* and its wild progenitor *L*. *orientalis* was difficult to distinguish due to this paraphyletic relationship. *L*. *culinaris* accessions ILL1704, IG 72622 and PI 572376 were located at this species boundary. In addition, IG 72611 was classified as *L*. *orientalis*, however, it was the earliest diverging member of the *L*. *orientalis*/*L*. *culinaris* clade. We conclude that these four samples are natural hybrids between *L*. *orientalis* and *L*. *culinaris* due to contradictory morphological and phylogenetic evidence.

Next, we examined the reproducibility of GBS results based on phylogenetic positioning since about 25% of the accessions had two or more biological replicates. Our hypothesis was that biological replicates of the same accession should be closely related and shared minimal genetic distance with each other. We found that the biological replicates of accessions from *L*. *culinaris* and *L*. *orientalis* were closely grouped together, however, this was not observed in other species especially in *L*. *nigricans*, *L*. *ervoides* and *L*. *lamottei* where an accession was more closely related to other accessions from the same GBS run than to their biological replicates from a different GBS run. This suggested that run-to-run variation was present in most wild lentils accessions and had a profound effect on the groupings of accessions and their biological replicates.

**Fig 3:**
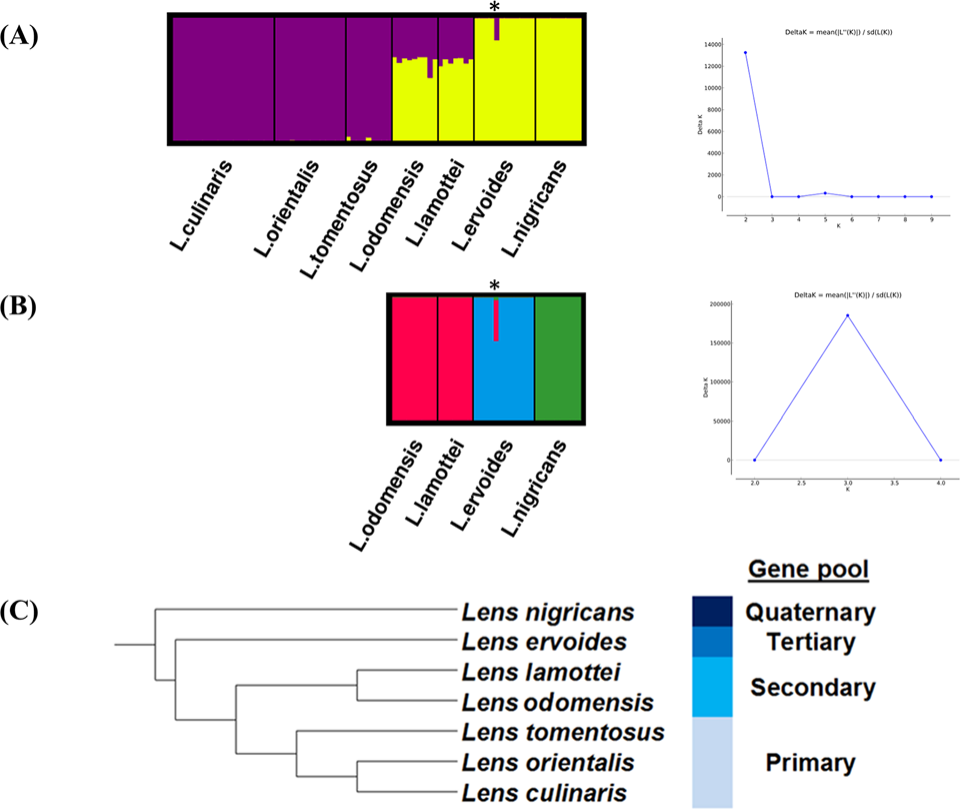
Gene pool classification of lentil based on STRUCTURE results. (A) Graphical representation of STRUCTURE results indicates two clusters (K = 2) in genus *Lens* based on the highest delta K score. (B) Additional STRUCTURE analysis revealed substructures (K = 3) within individuals of *L*. *odemensis*, *L*. *lamottei*, *L*. *ervoides* and *L*. *nigricans*. (C) The new gene pool classification proposed in this study is shown next to a simplified maximum-likelihood tree of genus *Lens*. Accession IG 72815 is marked with asterisk.

The Bayesian STRUCTURE analysis based on all individuals (Fig 3A) led to the observation that *L*. *culinaris/L*. *orientalis/L*. *tomentosus* and *L*. *ervoides*/*L*. *nigricans* each belong to one cluster while *L*. *lamottei* and *L*. *odemensis* showed mixed ancestry with major proportion in *L*. *ervoides*/*L*. *nigricans* cluster. This finding was in agreement with phylogenetic analysis except the fact that *L*. *ervoides* and *L*. *nigricans* were placed in the same cluster. The failure to reveal strong level of genetic divergence between two distinct populations suggested that hierarchical clustering plays a role in masking population substructure as reported by [68]. Additional STRUCTURE analysis using only individuals from *L*. *odemensis*, *L*. *lamottei*, *L*. *ervoides* and *L*. *nigricans* indicated three population substructures within these four species (Fig 3B). *L*. *odemensis* and *L*. *lamottei* belonged to one cluster while *L*. *ervoides* and *L*. *nigricans* each formed a distinct cluster. Based on these findings, we proposed a new gene pool classification for the genus *Lens* (Fig 3C). Both analyses revealed potential admixture between populations in one *L*. *ervoides* accession (IG 72815). This finding was consistent with the observation that IG 72815 shared shorter genetic distance compared to other accessions from the same species in the phylogenetic tree. Since this accession is from multiple plants, it is not clear if the observed admixture occurred due to natural hybridization or contaminated seed sources from another species.

Lastly, we re-examined the authenticity of our existing germplasm collection based on results from both GBS runs and identified 17% misclassified samples (See S1 Table). In the first GBS run, we identified discrepancies in taxon classification between genebank records and the phylogenetic tree in four samples and subsequently, re-sequenced those samples from a different seed source. Results from the second GBS run confirmed that the taxon classification based on the first phylogenetic tree was correct and it is consistent with the evidence based on morphological traits and crossability of mapping populations. We also identified four mislabeled samples which were likely caused by human error during sample and library preparation. One sample of IG 72613 was mislabeled as IG 72623, IG 72534 was suspected to be IG 72543 while two samples of IG 72529 were mislabeled as IG 72525. Besides identifying mislabeled accessions, we were able to validate the authenticity of some accessions and characterize the genetic differences within the same accession. One example is *L*. *orientalis* IG 72643. Our germplasm collection contains several seed sources of IG 72643 including one seed source which had red cotyledons (IG_72643_Sr2) instead of the expected yellow cotyledons. Our observation that all the seed sources formed a clade with minimal genetic distance in the phylogenetic tree suggested that they were authentic and no mislabeling or contamination has occurred and rather there is residual genetic variability within this accession.

## Discussion

Our comparison of one-enzyme vs. two-enzyme approach using a draft lentil genome assembly suggested that the two-enzyme approach can reduce overall genome complexity more than the single-cutter approach. Even genome distribution of GBS SNPs was observed based on the *M*. *truncatula* genome suggested that the SNPs are likely distributed evenly across the lentil genome as the genomes of both species share high levels of conserved synteny[69]. Since an automated GBS pipeline to map paired-end reads was not available at the start of this study, we developed a pipeline to reproduce the workflow from processing of raw reads through to SNP calling based on commonly-used open-source bioinformatics tools such as Bowtie2[49] and Samtools[50]. This enabled us to combine and re-use previously written codes to analyze our GBS data. A total of four commands are called to process raw Illumina paired-end reads through de-multiplexing, trimming and filtering, alignment to a reference genome and SNP calling. Our pipeline has several advantages compared to other GBS pipelines such as Tassel-GBS v3.0[70] and its reference-free pipeline UNEAK[71]. Our pipeline allows the paired-end reads to be more accurately mapped to a reference genome based on expected fragment sizes and has no upper limit on read length compared to the single-end mode and 64 bp tag length limitation in Tassel-GBS v3.0 and UNEAK. Our pipeline allowed us to simplify customization and alteration of all parameters from barcode sequence to mapping and variant calling parameters for future analysis while also providing a simplified user interface for more standardized runs. Through automatic generation of summaries at the end of each step, the user is given the opportunity to evaluate data integrity and tweak parameters accordingly.

Our assessment of technical reproducibility of GBS results identified several factors affecting the reproducibility which were not reported in previous GBS studies. One major problem with GBS for large genomes is low coverage sequencing data resulting in large numbers of missing data and false positive SNP calls. Our results suggested that the quality of the DNA samples is likely to be the main determinant of the number of sequencing reads. Firstly, sequencing bias is observed between samples but not found between technical replicates of a given sample, suggesting that sequencing bias is sample dependent. Assuming minimal methodological and sequencing machine variation, this bias is likely to be caused by DNA quality. This is not surprising as it is generally known that DNA quality affects digestion of restriction enzymes and ligation of adaptors. PCR amplification further escalates sequencing bias resulting in overrepresentation of PCR fragments from good quality samples. Therefore, we recommend paying particular attention to the quality of the DNA samples to ensure even sequencing coverage of a GBS run. Other known factors causing sequencing bias are fragment length bias, GC bias, unequal pooling of primers and DNA samples in multiplex reaction [72, 73], any or all of which may also have played a role in the uneven sequence coverage.

Our observation that an accession shared minimal genetic distance with other accessions from the same run in the final phylogenetic tree compared to its biological replicates of a different run suggested that run-to-run variation affects GBS results. This is not surprising as run-to-run variation has already been reported [74, 75]. One explanation is that run-to-run variation is caused by low sequencing coverage resulting in non-uniform sequencing of genomic regions between runs and thus, different SNPs are detected and used in phylogenetic tree construction. To reduce this effect, repeat sequencing with selected samples and increasing sequencing coverage has been recommended [74]. Interestingly, the effect of run-to-run variation was observed in all lentil samples except those of *L*. *culinaris* and *L*. *orientalis*, suggesting that ascertainment bias is likely playing a role. It is generally accepted that GBS eliminates most sources of marker ascertainment biases by discovering and genotyping markers simultaneously [34]. However, ascertainment bias can be re-introduced by using a reference genome from one species to map reads from its relatives. This can result in reduced read mapping and subsequent inability to detect rare alleles from genomic regions which are absent in the reference genome due to structural variation and differences in genome content. The inability to capture the real level of variation among individuals of a wild species made it appear as if there is very little genetic variability within and between species. To more accurately reflect the diversity within a wild species, it would be necessary to either carry out a *de novo* assembly or non-reference mapping based on the results of individual species. Either of these options would require additional sequencing to increase the depth of coverage sufficiently, which would add to the cost considerably. If the goal is simply to classify accessions to species, however, this additional work is not necessary.

Using phylogenetic and population structure analysis based on 5,389 good quality and non-redundant SNPs, we propose that the genus *Lens* should be separated into four gene pools, namely *L*. *culinaris/L*. *orientalis/L*. *tomentosus*, *L*. *lamottei*/*L*. *odemensis*, *L*. *ervoides* and *L*. *nigricans*. There is no doubt that *L*. *tomentosus* and *L*. *orientalis* belong to the primary gene pool as they are most closely related to *L*. *culinaris* and they can easily produce seeds following interspecific crossing. The second most closely related group to *L*. *culinaris* is *L*. *lamottei*/*L*. *odemensis*, placing them in the secondary gene pool. Our observation that *L*. *odemensis* is a sister clade to *L*. *lamottei* is in agreement with Verma et al. [76]. We propose that *L*. *odemensis* should be a separate species instead of a subspecies under *L*. *culinaris* as it is clearly distinct from the primary gene pool. As was suggested by Fratini and Ruiz [29], *L*. *ervoides* belongs to the tertiary gene pool as evidenced by the successful development of RIL mapping populations following F_1_ embryo rescue [17, 66]. Considering that interspecific crosses between *L*. *culinaris* and *L*. *nigricans* have never been reported to be successful beyond the F_1_ generation and both species formed distinct groups as revealed by phylogenetic and STRUCTURE analysis, *L*. *nigricans* should be placed in the quaternary gene pool and probably should be considered a last resort as a source of genetic diversity for cultivated lentil.

These results demonstrate several uses of GBS in the characterization of diverse germplasm. Firstly, GBS can be used as a reliable screening tool for lentil breeders interested in using wild relatives as a source of genetic diversity. GBS results provide good classification at the taxon level. Basing SNPs on comparisons with cultivated lentil however, results in the discriminatory power within species being limited to only *L*. *culinaris* and *L*. *orientalis*. Therefore, the use of GBS to correct identification of genotypes within wild species is not recommended until further optimization and improvement can be made. Secondly, GBS is an affordable screening tool replacing the need for other technology by discovering and genotyping many markers at once. We estimated that the cost per sample in this study for 48 samples in a 96-plex GBS run is CAD $48. The decreasing cost of sequencing is expected to drive cost per sample below USD $10 [34]. The low cost makes it feasible and practical to screen wild germplasm accessions before use in introgression. This cost would offset the potential larger cost of making a mistake in crossing due to mis-classification. Lastly, GBS is useful for checking the authenticity of germplasm. This has been demonstrated in Labate et al. [43] where a *Solanum arcanum* accession was re-classified as *S*. *huaylasense* based on GBS data. A previous diversity study in lentil germplasm [77] re-classified 10.8% of *L*. *culinaris* and *L*. *orientalis* germplasm based on phylogenetic tree results suggesting the need to examine the authenticity of lentil germplasm before utilization. Marker information can be used to detect errors in germplasm collections, resulting in more time and resources devoted to varietal development with a higher success rate.

In summary, GBS is a promising technology for plant breeders interested to work with crop wild relatives as an affordable and reliable routine screening of germplasm provided that several technical problems are addressed. The reliability and practically of GBS is likely to increase in the future with the improvement of sample preparation, increased sequencing depth, reduced sequencing cost per base and *de novo* sequence assembly of wild relatives. This technology offers high potential for screening largely uncharacterized gene pools of non-model crops with few genomic resources.

## Supporting Information

S1 Table Detailed information of accessions used in genotyped-by-sequencing. (XLS)

S2 Table Summary of genotyping-by-sequencing including number of reads, mappability and expected site coverage. (XLS)

S3 Table Distance matrix and Newick format of phylogenetic trees. (XLS)
